# Observation on the Visibility Decrease in a VCN Spin Resonator Interferometry

**DOI:** 10.6028/jres.110.033

**Published:** 2005-06-01

**Authors:** M. Utsuro, M. Hino, P. Geltenbort, J. Butterworth

**Affiliations:** Research Center for Nuclear Physics, Osaka University, Osaka, Japan; Research Reactor Institute, Kyoto University, Kyoto, Japan; Institut Laue-Langevin, Grenoble, France

**Keywords:** interference visibility, Larmor precession, multilayer resonator, neutron spin echo, spin interference, tunnelling transmission, ultracold neutron anomaly, very cold neutrons

## Abstract

The present paper reports on the detailed studies concerning the neutron spin interference visibility observed after transmitting through multilayer magnetic resonators in a spin echo condition with very cold neutrons from a high flux reactor. The observed visibility of the interference between upward and downward spin components perpendicular to the Larmor precession plane of the neutron spin are compared with the numerical simulations in the plane wave theory and also in the Schrödinger wave-packet model. The comparison revealed the instructive characteristic features of obvious additional visibility decrease observed in the interference between the tunnelling and refractive transmissions of each spin components in a single as well as a couple of multilayer magnetic resonators.

## 1. Introduction

There are several reports on the ultracold neutron anomalies observed in the neutron bottle experiments in which ultracold neutrons (UCN) with the velocity below about 6 m/s were stored for a long time, experiencing total reflections even at normal incidence to the material mirror surface composing the closed vessel. For example, recent experiments on beryllium coated bottles with various surface cleaning treatments [1] gave a high precision value for the reflection loss probability of UCN per reflection. This indicates an anomalously higher value at the extrapolation to the lowest temperature ends than those from the neutron transmission experiments and also from the theoretical cross section for a clean sample of beryllium. Another recent study on the leakage of UCN [2] from a bottle showed the subcritical energy neutrons anomalously transmitted through an aluminum window, in contradiction to the simple expectation from plane wave theory that predicts essentially no transmission. Therefore, we think further detailed experiments are necessary to study the subcritical transmission phenomena of long wavelength neutrons through a material layer.

## 2. Experimental Scheme and Arrangements

Since the observed reflection and transmission anomalies are supposed to be characteristic features in long wavelength neutrons, our present experiments are carried out with the very cold neutrons (VCN) of about 6 nm extracted from the high flux reactor at the Institut Laue-Langevin (ILL). As the instrument for elucidating the details of the wave phenomena, a spin interferometer device [3] especially arranged at the PF2-VCN port of the reactor was used. This was because the tunnelling and refractive transmissions of parallel and antiparallel spin neutrons through a magnetic layer are the completely same quantum optical phenomena as those occurring in the rigorous solutions of the wave equation when applied to the neutron experiments mentioned in the previous section.

For enhancing wave effects in the transmissions phenomena in the spin echo interferometry, two exactly equivalent high-layer number Fabry-Perot magnetic resonators were specially prepared [4] with alternative evaporation of magnetic (15 nm thick Parmalloy) tunnelling layers and non-magnetic (40 nm thick germanium) gap layers, totaling 21 layers on a silicon substrate.

**Fig. 1:**
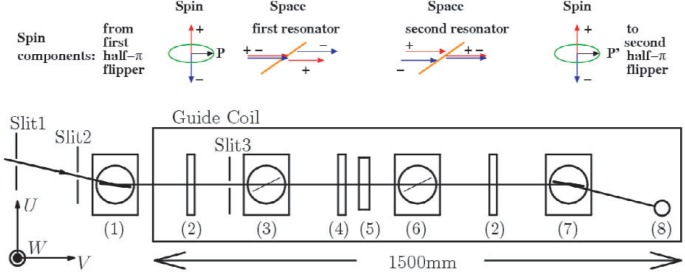
Schematic arrangement of the neutron spin interferometer and the spin components behaviours for two magnetic resonators inserted as the samples. (1) Monochromator-polarizer, (2) π/2 spin flipper coil, (3) first Fabry-Perot magnetic resonator (sample S1), (4) π spin flipper coil, (5) accelerator coil, (6) second Fabry-Perot magnetic resonator (sample S2), (7) analyzer, and (8) ^3^He detector.

The experimental setup and the spin component behaviours are shown in Fig. 1, where the VCN passing through two slits (Slit 1 and 2) with a separation of 1000 mm on both sides of an evacuated flight tube are monochromated and polarized. Then, with the spin half-π turned to lie on the horizontal plane (corresponding to the superposition of parallel and antiparallel components to the direction of the guide field), the neutrons pass through Slit 3 and enter the first resonator sample standing on a remote controlled high precision goniometer.

In the resonator sample, the parallel spin component feels a higher potential than the antiparallel component in the magnetic layers. Thus, the former experiences the tunnelling transmission while the latter experiences refractive transmission in a certain glancing angle region.

After the first resonator, each spin component is reversed, and then with the exact same compensating spin precessions through the second resonator, the second half-π flipper and the spin analyser exert to draw the neutron spin echo (NSE) pattern in the neutron count rates with the interference visibility. This occurs in the integration over the precession angle distributions for the finite wavelength resolution and the neutron angular divergence.

Prior to such twin-resonator measurements, similar measurements on a single resonator with only the first sample or the second one inserted were also carried out. These measurements were repeated during two reactor cycles in order to ensure the experimental reproducibility.

## 3. Numerical Simulation Approaches and the Parameter Values

**Fig. 2:**
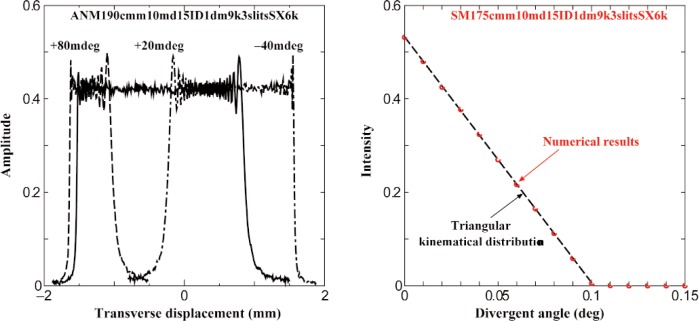
Typical results of numerical simulations at the first sample position on the plane wave interferences passing through the three serial slits in the present experimental configuration: transverse distributions of the wave amplitude for typical three kinds of incident angles (left); and the angular distribution of the total intensity compared with the simple kinematical approximation (right).

The spatial and angular distributions of the neutrons incident on the samples in view of the possible slit interferences through the three serial slits in Fig. 1 were numerically studied according to the plane waves superposition approach [5] for the divergent angular distribution of plane waves entering the Slit 1. Such coherent superpositions were performed successively over the same transverse widths (3.0 mm) of Slits 1 to 3, and the neutron waves at the sample positions were shown to be well described by the truncated plane waves as shown typically in Fig. 2 (left). Furthermore, with superpositions over the angular distribution of the initial incident plane waves and also over the wavelength resolution of the monochromator, the intensity distributions at the samples were obtained as those expressed very well by a simple kinematic approximation as shown in Fig. 2 (right). The effects of the transverse separation between the spin components described in Fig. 1 were also assured to be little even in the single resonator setup.

The incident neutron spectrum on the sample in the present VCN interferometer was already studied [3] with a chopper and time-of-flight method. The exact values for the average wavelength and the resolution, 5.7 nm and 10 %, respectively, were assured with satisfactory agreements between the measured transmission patterns and the calculations using the numerical simulation program [4].

The exact equality of two present resonators and the values for various parameters for the resonators and the interferometer to be employed in our simulations were assured comparing with the experimental results. Both of the resonator samples showed exactly the same values for the three resonant peaks observed in the angular plots of the transmission ratio: the lowest resonant angle at about 4.5° named subcritical resonance, the middle at about 6.8 degrees as transcritical, and the remaining at about 9° as supercritical, as compared to the parallel spin critical angle for total reflection being 6.32°.

For our interference simulations in the plane wave theory, we extended our previous program [4] prepared with the decomposition of the wave vector to the parallel and the perpendicular components to the sample surface. For our high-layer number magnetic resonators and making use of the transfer matrix technique [6], we now take into consideration the transverse angular divergence of the incident neutrons at the resonator samples in addition to the effects of the wavelength resolution.

Furthermore, we extended our simulation program to the Schrödinger wave-packet approach [7,8,9] with the coherent superpositions over the packet wavelength width and the transverse angular divergence of the incident neutrons to the samples. As the supposed magnitudes for these parameters, various values were studied for the packet wavelength width, while the transverse angular divergence was decided from the result shown in Fig. 2.

In these simulations, the possible very small angular and magnetic discrepancies between twin samples were also considered.

## 4. Derivation of Experimental Interference Visibility

A new method was employed in our present spin interferometry. This is in contrast to the conventional spin echo method of using an accelerator coil, what we call the π flipper phase scan [10] with the three RF flippers setup. In other words, we scan the NSE signal, changing the phase of the oscillating field in the π flipper coil independently to those of the two π/2 flipper coils operated completely in a same phase. Thus, we employ the phased synchronous oscillations of these three flipper coils at an exactly same frequency. In our method of the π flipper phase scan with the three RF flippers setup, the resultant NSE pattern could be derived as one period of the π flipper signal corresponding to two turns of the Larmor precession of neutrons in the apparatus [10].

We define the visibility of the spin interference as the ratio between the interference amplitude and the mean level of the neutron count rates. These values are obtained from the sinusoidal curve fitting to the measured count rate patterns after background subtraction. Furthermore, for extracting the effects of the resonator samples from the measured visibility, we assume that the net visibility due to the resonator samples is simply given by the observed visibility divided by the visibility without the samples. We obtained about 60 % as the value for the no-sample visibility from the sinusoidal fitting and background subtraction.

**Fig. 3:**
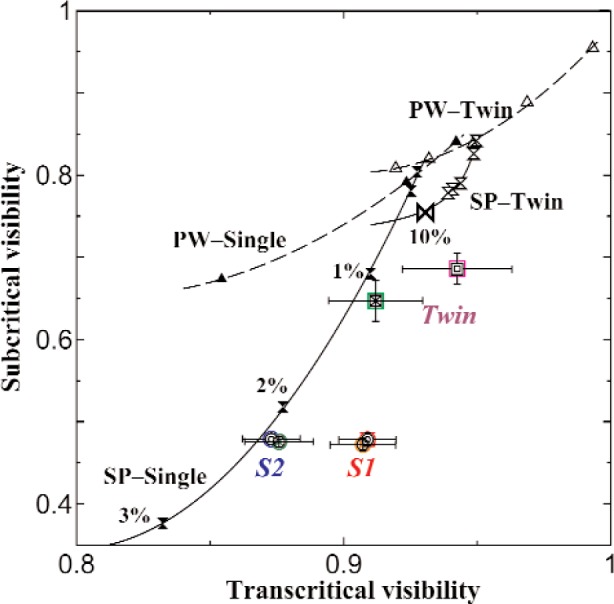
Experimental results of subcritical and transcritical visibilities on single (S1 and S2) and twin resonators compared with the plane wave theory (PW) and also with the Schrödinger wave-packet model (SP) with the values for packet wavelength width indicated.

In the case of twin-resonator measurements, a more laborious procedure than the single resonator case must be employed for attaining the complete parallel condition between twin samples. In other words, one scans the second sample angle with a small angle step to a fixed first sample angle and then performs a Gaussian fit for such a visibility scan observed in order to derive the visibility value for the exact parallelism. Each result of the visibility decrease obtained from the twice-repeated measurements are shown in Fig. 3 and compared with the numerical simulations based on the theories. The error bars show the root squared sum of the standard deviation from the sinusoidal fitting and the deviation of the Gaussian peak from the nearest experimental point in the case of twin resonators.

## 5. Discussions and Concluding Remarks

The present experiments of the VCN spin resonator interferometry gave the interference visibility with satisfactory reproducibility for each of single and twin resonator setups. The simulation calculations indicated the general correlation tendencies between the subcritical and transcritical visibilities. The plane wave simulations showed significant discrepancies in the subcritical visibilities from the measured results both in the single and twin resonator setups. The numerical simulations in the Schrödinger wave packet model also indicated obvious discrepancy remaining for the twin resonator case and some difference in the tendency for the single resonator case. The present discrepancies in the subcritical visibilities suggest there exists some cause of additional precession phase distribution broadening in the tunnelling transmission not considered in the present simulations and induced the measured visibility decrease after the superpositions over the phase distributions in the measuring process.
